# Including non-concurrent control patients in the analysis of platform trials: is it worth it?

**DOI:** 10.1186/s12874-020-01043-6

**Published:** 2020-06-24

**Authors:** Kim May Lee, James Wason

**Affiliations:** 1 MRC Biostatistics Unit, School of Clinical Medicine, University of Cambridge, Cambridge Institute of Public Health, Forvie Site, Robinson Way, Cambridge Biomedical Campus, Cambridge, CB2 0SR UK; 2 Population Health Sciences Institute, Newcastle University, Baddiley-Clark Building, Newcastle University Richardson Road, Newcastle upon Tyne, Newcastle upon Tyne, UK

**Keywords:** Adding arms, Concurrent/ non-concurrent control, Platform trials

## Abstract

**Background:**

Platform trials allow adding new experimental treatments to an on-going trial. This feature is attractive to practitioners due to improved efficiency. Nevertheless, the operating characteristics of a trial that adds arms have not been well-studied. One controversy is whether just the concurrent control data (i.e. of patients who are recruited after a new arm is added) should be used in the analysis of the newly added treatment(s), or all control data (i.e. non-concurrent and concurrent).

**Methods:**

We investigate the benefits and drawbacks of using non-concurrent control data within a two-stage setting. We perform simulation studies to explore the impact of a linear and a step trend on the inference of the trial. We compare several analysis approaches when one includes all the control data or only concurrent control data in the analysis of the newly added treatment.

**Results:**

When there is a positive trend and all the control data are used, the marginal power of rejecting the corresponding hypothesis and the type one error rate can be higher than the nominal value. A model-based approach adjusting for a stage effect is equivalent to using concurrent control data; an adjustment with a linear term may not guarantee valid inference when there is a non-linear trend.

**Conclusions:**

If strict error rate control is required then non-concurrent control data should not be used; otherwise it may be beneficial if the trend is sufficiently small. On the other hand, the root mean squared error of the estimated treatment effect can be improved through using non-concurrent control data.

## Background

The implementation of innovative trial designs for drug development programmes has increased in recent years [1]. A platform trial is one such innovative design that helps improve efficiency. It has a multi-arm multi-stage design where a single control group is utilized to evaluate multiple experimental treatments for a single disease. Apart from the feature of dropping arms, a platform trial allows adding arms when new experimental treatments become available. This adds efficiency as adding arms takes less time and has a lower cost than setting up new trials from scratch.

A platform trial can be implemented through the specification of a master protocol [2–4]. Cohen *et al* [5] review the statistical aspects on the adding arm adaptation and present some trials that have adopted a platform approach. For pairwise comparisons between the effectiveness of newly added treatments and the control treatment, there are different views on utilising the control data [5]. To be more specific, the control data of a platform trial can be separated into non-concurrent and concurrent. The former (latter) corresponds to the control data of the patients who are recruited before (after) a new experimental treatment is added. This definition applies to each newly added arm independently; the control data can be concurrent to a newly added treatment, and be non-concurrent to another treatment that is added at a later time point.

To our knowledge, methodology and guidance for adding arms remain limited and the operating characteristics of platform trials have not been well-studied. The literature on other trial adaptations such as dropping arms and changing treatment allocation ratio are more comprehensive [6*–*11]. In particular, many have explored the presence of time trends when more complex randomization procedures are recommended/ explored [12*–*21]. A trend may be present in a trial when there is a learning curve among the study personnel; a shift in the population baseline characteristics; and/ or the effect of the control treatment may change due to other reasons (e.g. improvement of the standard of care/ practice) [7,22].

In order to minimise the impact caused by the presence of a trend, most researchers advocate the use of concurrent control data when making inference about the newly added treatment(s) [23*–*25]. Others stipulate that non-concurrent control data may be used when making inference about the newly added arm by adjusting for possible trends [26,27]. Here we investigate whether using non-concurrent control data is worthwhile in platform trials. Several analysis approaches are considered. Within a two-stage setting where a new treatment is added at the end of stage one [25,28], we explore the impact of i) the timing of adding a new arm ii) the sample size of the new arm and iii) the magnitude of a linear or a step trend [15] on the inference about the newly added experimental treatment. We provide recommendations about under which conditions non-concurrent control data should be used.

## Method

Consider a two-stage multi-arm trial that initially has *K* treatments and a control treatment with a total sample size of *N*=*N*_1_+*N*_2_ where *N*_*s*_ is stage *s* sample size, *s*=1,2. Denote *k*=0 for a control treatment, *k*=1,...,*K* for the initial experimental treatments. Let *n*_*ks*_ be the sample size of treatment *k* in stage *s* with *n*_*k*1_+*n*_*k*2_=*n*_*k*_. At the end of stage one, in particular after $N_{1}=\sum _{k=0}^{K} n_{k1}$ patients have been randomized to the initial arms in stage one, a new treatment (denoted by *K*+1) is added to the on-going trial with *n*_*K*+1,2_ patients. This increases the second stage sample size to *N*_2_+*n*_*K*+1,2_ and the overall sample size of the trial to *N*+*n*_*K*+1,2_.

Denote *n*_01_/*n*_0_ as the timing of adding a new arm. Small *n*_01_/*n*_0_ indicates the newly available treatment is added to the trial after a small number of patients have been randomized to the initial arms in stage one. We consider the case that all arms finish recruitment simultaneously (although as long as controls continue to be recruited while treatment *K*+1 is allocated patients, results apply more generally). For *j*=1,...,*N*+*n*_*K*+1,2_, denoting the sequence of patient enrolment to the trial, let:

1 $$ {X}_{j \ }= { \sum_{k=0}^{K+1} \mu_{k} \cdot I(T_{j} =k) }+ \tau(j) + \epsilon_{j}   $$

be the response of treatment *k* of subject *j*; *μ*_*k*_ be the true effect of treatment *k*; *τ*(*j*) be a trend that could be a function of the patient ordering *j*; *ε*_*j*_ be the random errors that are identically and independently normally distributed with mean 0 and variance *σ*^2^; and *T*_*j*_ be the allocated treatment of subject *j*, with *I*(·) is an indicator function.

Let $\bar {X}_{k.s}$ and $\bar {X}_{k}$ denote the stage *s* sample mean response of treatment *k* and the overall mean response of treatment *k*. When there is no trend in the trial, i.e. *τ*(*j*)=0(*j*), the sample mean estimators $\bar {X}_{k.s}\overset {iid}{\sim } N(\mu _{k}, \sigma ^{2}/n_{ks})$ and $\bar {X}_{k}\overset {iid}{\sim } N(\mu _{k}, \sigma ^{2}/n_{k})$.

For illustration purpose, we consider the following when exploring the impact of a fixed trend on the inference:

- linear trend:
  2 $$\begin{array}{*{20}l} \tau(j)= \lambda \ (j -1) / (N+n_{K+1,2}-1)  \end{array} $$
- stepwise trend:
  3 $$\begin{array}{*{20}l} \tau(j)= \lambda \cdot c_{j}  \end{array} $$

where *c*_*j*_ is an indicator that subject *j* is enrolled in stage two, and *λ* is a positive fixed value. Both of these trends inflate the value of the outcome variable in a sequential manner; the responses of the patients who were enrolled later are larger than those who were enrolled earlier.

### Simple approach: *Z*-test

We first consider a simple method that is analogous to ignoring the possibility of a trend or stage effect: a standard *Z*-test for testing each hypothesis

**Table:** 

*H*_0*k*_:*μ*_*k*_=*μ*_0_	against	*H*_1*k*_:*μ*_*k*_>*μ*_0_,

using the sample means. When there is no trend, the (pairwise) marginal power of rejecting a null hypothesis to detect a difference in treatment effect, *δ*_*k*_=*μ*_*k*_−*μ*_0_, is

$$\Phi \left[ \frac{ \delta_{k}}{\sqrt{\sigma^{2}/ n_{k} + \sigma^{2}/ n_{0}}} - \Phi^{-1}(1-\alpha) \right], $$

where *Φ* and *Φ*^−1^ are the cumulative density function and the inverse of the cumulative density function of a standard normal distribution. For the newly added treatment, the power of rejecting *H*_0,*K*+1_ depends on whether all the control data (of size *n*_0_) or only the stage two control data (of size *n*_02_) is used in the inference. Elm *et al* [28] describe a *t*-test for the case where *σ*^2^ is unknown.

### Model-based approach: weighted linear regression

One way to account for the changes of a trial design is to consider a model-based approach. Elm *et al* [28] consider the following weighted linear regression model. Let $\bar {X}^{(k)}_{s}= \bar {X}_{k.s}- \bar {X}_{0.s}$ be the stage-wise difference in mean responses; fit a linear model to the samples of the stage-wise mean differences:

$$\bar{X}^{(k)}_{s}=\delta_{k} + \eta^{(k)}_{s} $$

where $\eta ^{(k)}_{s}$ is normally distributed with mean zero and variance *σ*^2^/*n*_*ks*_+*σ*^2^/*n*_0*s*_. The covariance between mean responses, $cov(\bar {X}^{(l)}_{1},\bar {X}^{(k)}_{2})=0 \forall l$ and *k*=1,...,*K*, as patient data are independent; whereas $cov(\bar {X}^{(l)}_{s},\bar {X}^{(k)}_{s})= \sigma ^{2}/ n_{0s}, \forall l \neq k$, where *l*,*k*=1,...,*K*, as the data of a shared control group is used in all the pairwise comparisons. For the newly added treatment, $cov(\bar {X}^{(K+1)}_{2}, \bar {X}^{(k)}_{1})= 0$ and $cov(\bar {X}^{(K+1)}_{2}, \bar {X}^{(k)}_{2})= \sigma ^{2}/ n_{02}$ for *k*=1,...,*K*, when only the concurrent control data is used.

Elm *et al* [28] have not considered using all the control data in estimating the difference in mean responses of treatment *K*+1 and the control, i.e. defined as $\bar {X}^{(K+1)'}_{2}= \bar {X}_{K+1,2}- \bar {X}_{0}$. This estimate has a smaller variance than $\bar {X}^{(K+1)}_{2}$, and $cov(\bar {X}^{(K+1)'}_{2}, \bar {X}^{(k)}_{s})= \sigma ^{2}/ n_{0}$ for *k*=1,...,*K*, and *s*=1,2, as $\bar {X}_{0}$ can be re-expressed as $\frac {n_{01}}{ n_{0}} \bar {X}_{0.1}+ \frac {n_{02}}{ n_{0}} \bar {X}_{0.2}$.

Using weighted least squares estimation, an estimate of the vector of treatment effects relative to control, ***δ***=(*δ*_1_,...,*δ*_*K*_,*δ*_*K*+1_)^*T*^, can be obtained. The estimate, $\boldsymbol {\hat {\delta }}$, is unbiased and has a multivariate normal distribution with mean ***δ*** when there is no trend. This joint distribution can be used to test the hypotheses, {*H*_01_,...,*H*_0,*K*+1_}, in a similar way to the Dunnett test [29]. The marginal power of rejecting a hypothesis can be computed by considering the corresponding marginal distribution. Note that when *K*>0, the marginal power is different to the power obtained from a *Z*-test (or a *t*-test) as the joint distribution accounts for the fact that a common control group is used in the analysis. Note also that $var(\hat {\delta }_{K+1})$ depends on the number of control patients.

We label these approaches as *WLS*_*all*_ and *WLS*_*s*2_ respectively, when all the control data and when only the concurrent control data are used in the estimation of *δ*_*K*+1_.

### Model-based approach: linear regression

In the context of an adaptive randomisation procedure, Villar et al. [19] considered fitting a linear regression model to adjust for the presence of a trend in their investigation where all arms start recruiting at the same time. We explore the following linear regression analyses for the trial that adds an arm at the end of stage one:

- *M*_*a*1_: $X_{j }= \alpha + { \sum _{k=0}^{K+1} \beta _{k}\cdot I(T_{j}=k) }+ { \gamma } \cdot j + \epsilon _{j}$
- *M*_*a*2_: $X_{j }=\alpha + { \sum _{k=0}^{K+1} \beta _{k}\cdot I(T_{j}=k)} + { \nu } \cdot c_{j}+ \epsilon _{j}$

where *β*_0_=0 such that *β*_*k*_ represents the difference in mean responses of treatment *k* and the control treatment; and

- *M*_*b*1_: $X_{j }=\alpha + {\beta _{0} \cdot I(T_{j}=0)} + \beta ^{b1}_{K+1}\cdot {I(T_{j}=K+1)} + { \gamma } \cdot j + \epsilon _{j}$
- *M*_*b*2_: $X_{j }=\alpha + {\beta _{0} \cdot I(T_{j}=0)} + \beta ^{b2}_{K+1}\cdot {I(T_{j}=K+1)} + { \nu } \cdot c_{j}+ \epsilon _{j}$

where *β*_0_=0 such that $\beta ^{b1}_{K+1}$ and $\beta ^{b2}_{K+1}$ represent the difference in mean responses of the newly added treatment and the control treatment. Note that models *M*_*a*1_ and *M*_*a*2_ use all data to estimates all *β*_1_,...,*β*_*K*_,*β*_*K*+1_, whereas model *M*_*b*1_ (and *M*_*b*2_) uses the data of the control arm (both stages) and the newly added arm to estimate $\beta ^{b1}_{K+1}$ (and $\beta ^{b2}_{K+1}$) only. The parameter *γ* in *M*_*a*1_ and *M*_*b*1_ represents the effect of continuous recruitment, whereas *ν* in *M*_*a*2_ and *M*_*b*2_ represents the effect of recruitment that happens after the new treatment has been added (Elm *et al* [28] interpret *ν* as a stage effect). It is worth mentioning that the amount of the control data used to estimate *β*_*K*+1_ in *M*_*a*2_ (and respectively $\beta ^{b2}_{K+1}$ in *M*_*b*2_) is similar to (same as) that of using only the second stage control data, e.g. $\bar {X}_{K+1,2}-\bar {X}_{0.2}$, since the newly added treatment was not present in stage one and the stage one control data would contribute to the estimation of the stage effect and that of *β*_1_,...,*β*_*K*_ under such a model set-up. In other words, the stage one control data contributes very little to the estimation of the difference in mean responses of the newly added treatment and the control treatment when we adjust for *ν*·*c*_*j*_ in the analysis.

We note that the inference from *M*_*a*1_ without adjustment for the linear term in patient ordering is similar to that from *WLS*_*all*_; and that from *M*_*a*2_ is similar to *WLS*_*s*2_. This is because the joint distribution of $\{\hat {\beta }_{1},..., \hat {\beta }_{K+1}\}$ is equivalent to the corresponding joint distribution of $\boldsymbol {\hat {\delta }}$. The subtle difference is the former approach has one more parameter (i.e. the intercept *α*) than the weighted regression model, which reflects the mean response of the reference group assuming the model is true. On the other hand, testing $\beta ^{b1}_{K+1}$ and $\beta ^{b2}_{K+1}$ respectively are similar to considering an independent *Z*-test (or a *t*-test when assuming unknown variance) for the hypothesis of *H*_0,*K*+1_.

### Metrics

We explore the above analysis approaches and consider

- bias of estimators;
- the type one error rate;
- the marginal power of rejecting a false null hypothesis;
- the root mean squared error,
  $${}\text{rMSE}=\sqrt{ \text{bias of an estimate}^{2}+ \text{variance of an estimate} };$$

when including or excluding the stage one control data in the analysis of the newly added treatment. As we assume *σ*^2^ is known, the presence of a trend would affect the accuracy of the estimate but not the variance of an estimate. We include the following metric to explore the reduction in the variance of the estimate:

$$BoS=\frac{V_{0}- V_{a}}{V_{0}}$$

where *BoS* stands for borrowing of strength [30], *V*_0_ and *V*_*a*_ correspond to the variance of an estimate that is computed with only the concurrent control data and that with all the control data respectively. Note that *BoS* increases as *V*_*a*_ decreases. This measure is similar to the *R*-squared that measures how close the data are to the fitted regression model. Here *BoS*≈0 indicates that the variability of the response data around its mean cannot be further explained by including non-concurrent control data; whereas *BoS*≈1 reflects that using all the control data results in a perfect estimate that has negligible variability. One may interpret BoS as an indicator of the benefit of including non-concurrent control data in the analysis of the newly added treatments. Small values show that including the non-concurrent control data provides little benefit to the estimation of the parameters in terms of precision gain. High value is desirable especially when the estimates of the variance are unbiased and are not affected by the presence of a trend.

In the next section, we consider *K*=1 and a new arm is added at the end of stage one. Let the effect size of the difference in treatment effects be *δ*_1_=*δ*_2_=0.15 under the alternative scenario, and *δ*_1_=*δ*_2_=0 under the null scenario, with *σ*^2^=1,*n*_11_=*n*_01_=*N*_1_/2, and *n*_12_=*n*_02_=*N*_2_/2. We choose *n*_0_=*n*_1_=550 to obtain 80% power and 5% type one error rate of rejecting *H*_01_ using the *Z*-test.

We conduct simulation studies to examine the impact of a trend with the timing of adding a new treatment, *n*_01_/*n*_0_={0.25,0.5,0.75}, and the sample size of the new arm, *n*_22_={*n*_02_,*n*_0_,2*n*_0_}. Following (1), we adjust the generated responses post randomization with *λ*={0.02,0.04,0.06,0.08} in the linear trend (2) and the stepwise trend (3) respectively. These values indicate a trend of *λ*×100*%* of standard deviation. Each scenario is replicated 100 000 times. We illustrate *n*_22_=2*n*_0_ as one of the options to explore the marginal benefit of having more patients (than necessary) in the newly added arm while keeping the size of the control arm the same as the initial plan.

## Results

### Analytical power and *BoS* when there is no trend

We now compare *BoS* and the power of rejecting *H*_02_ when there is no trend and either all, or only concurrent control data are used in the inference.

**Fig. 1:**
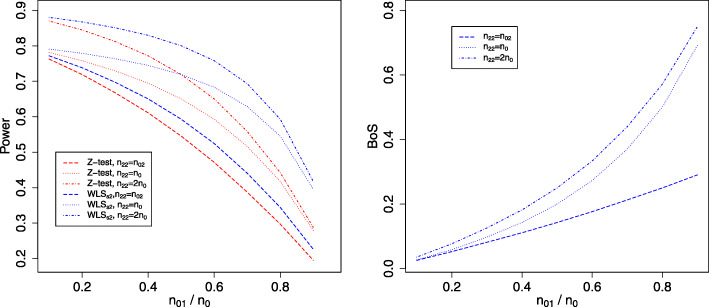
Left: power curves when the individual *Z*-test (red lines) and *WLS*_*s*2_ (blue lines) are used respectively for testing *H*_02_, both using stage two control responses. Right: reduction in $var(\hat {\delta }_{2})$ when stage one control responses is used relative to not using stage one control responses. Line types correspond to different values of *n*_22_ when the new arm is added to the on-going trial. The timing of adding the new arm is reflected by *n*_01_/*n*_0_ on the *x*-axis

**Fig. 2:**
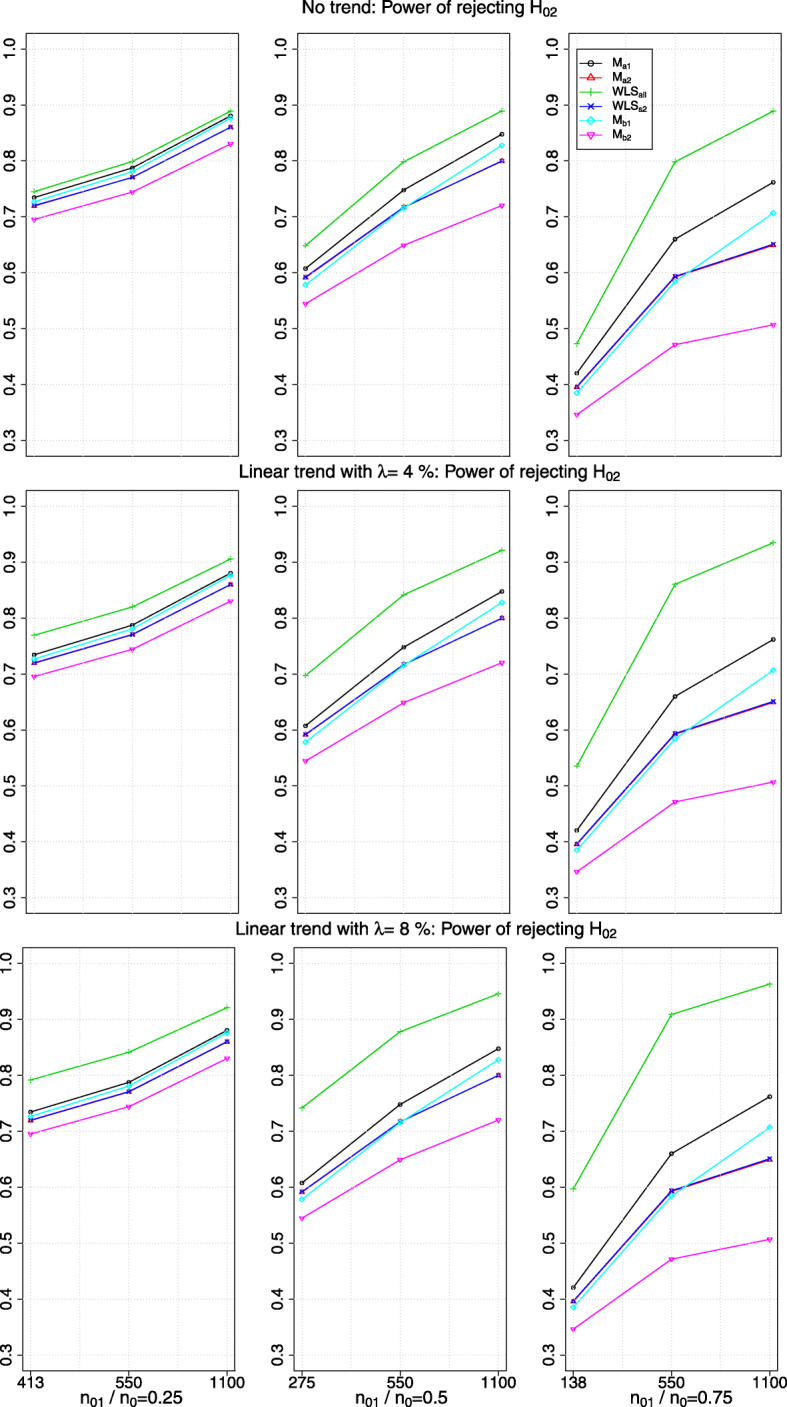
The power of rejecting *H*_02_ in the presence of no trend or a linear drift with a magnitude of *λ*>0 standard deviation (row-wise); *x*-axis indicates the value of *n*_22_ and the timing of adding the new arm

**Fig. 3:**
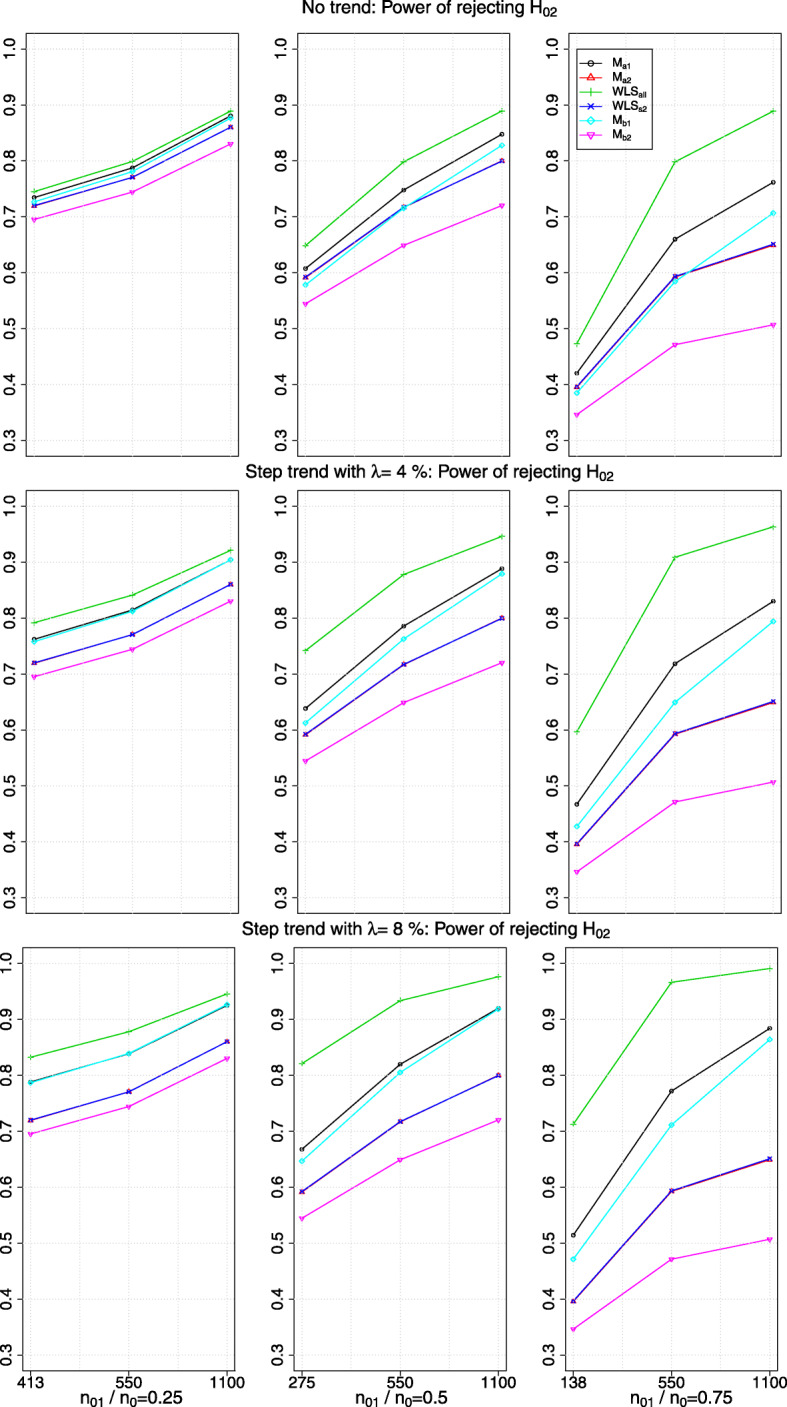
The power of rejecting *H*_02_ in the presence of no trend or a step trend with a magnitude of *λ*>0 standard deviation (row-wise); *x*-axis indicates the value of *n*_22_ and the timing of adding the new arm

We first demonstrate the differences between the independent *Z*-test (computed with only concurrent control data) and the model-based approach, *WLS*_*all*_ and *WLS*_*s*2_, in terms of power. For ease of presentation, we omit the comparisons to the presented linear regression models as they make adjustment for a trend (see the first row of plots in Figs. 2 and 3 for the corresponding marginal power). The left plot in Fig. 1 shows power curves when the individual *Z*-test (red lines) and *WLS*_*s*2_, i.e. the approach with the bivariate normal distribution of $(\hat {\delta }_{1}, \hat {\delta }_{2})$ (blue lines), are used respectively for testing *H*_02_, both using only the concurrent control responses. The timing of adding the new arm to the on-going trial is reflected on the *x*-axis; small values indicate the arm is added after a small number of patients have been randomized to the initial treatment arms. Different lines correspond to having different *n*_22_.

As expected, the power decreases with *n*_01_/*n*_0_ when excluding stage one control responses in the inference about the newly added treatment. Comparing the red lines to the blue lines in the left plot of Fig. 1, the power of the individual *Z*-test is lower than the marginal power obtained from *WLS*_*s*2_ given the same *n*_22_. When *n*_22_=*n*_02_, the difference between the power curves computed by the two approaches is less than 6% (shown by comparing the dashed lines). When *n*_22_≥*n*_0_, the magnitudes of the differences between the power curves are larger especially for large *n*_01_/*n*_0_; e.g. compare the dotted-dashed lines at *n*_01_/*n*_0_=0.8, the marginal power obtained from *WLS*_*s*2_ is 15% more than that computed from the individual *Z*-test. This finding highlights that considering the joint outcomes of the hypotheses through the joint distribution of the parameters is more efficient than using the individual *Z*-test when only the concurrent control data is used. This is because the former accounts for using a shared control group in the inference, whereas the *Z*-test uses the control data independently for each hypothesis test as if they were from several trials. Besides that, compare the (vertical) difference between the dotted-dashed line (*n*_22_=2*n*_0_) and the dotted line (*n*_22_=*n*_0_), either red or blue, we see that the magnitude of the difference in the power decreases as *n*_01_/*n*_0_ increases. This indicates that the marginal benefit of having a larger sample size in one arm than necessary can be small, depending on when the new arm is added to the trial.

For *WLS*_*all*_ with *n*_22_=*n*_02_, the power curve coincides with the red dotted line, i.e. the individual *Z*-test using only the concurrent control data and with *n*_22_=*n*_0_. This is not surprising as the same number of patients are used in the inference, albeit the number of patients randomized to the newly added arm is different for the two comparators (i.e. *WLS*_*all*_ considers *n*_02_ responses of the new treatment and *n*_0_ responses of the control treatment; the individual *Z*-test uses *n*_0_ responses of the new treatment and *n*_02_ responses of the stage two control treatment). For *WLS*_*all*_ with *n*_22_=*n*_0_ and *n*_22_=2*n*_0_, the marginal power of rejecting *H*_02_ is 0.8 and 0.89 respectively across all *n*_01_/*n*_0_, which is close to the respective power obtained from *Z*-test using all the control data.

We now consider the *BoS* values that are computed with $var(\hat {\delta }_{2})$ obtained from *WLS*_*all*_ and *WLS*_*s*2_ respectively. The right plot in Fig. 1 shows that when all the control data is included in the estimation, *BoS* increases with *n*_01_/*n*_0_. When *n*_22_=*n*_02_, *BoS* is close to 30% when the new treatment is added at *n*_01_/*n*_0_=0.9. When *n*_22_ has the same number as *n*_0_, we see that *BoS* becomes even larger. However, the marginal return of having large *n*_22_ than necessary can be less notable: comparing the dotted-dashed line to the dotted line shows that the magnitudes of the differences between the *BoS* perhaps are less impressive compared to the likely cost of enrolling an extra *n*_0_ patients for the new arm.

### Bias of the estimate when there is a trend

We now present the simulation findings of the inference about the newly added treatment when there is a trend.

Consider the estimated difference in mean responses of the newly added treatment and the control treatment. Table 1 shows the maximum absolute bias (and median absolute bias) in the parameter estimations when there is a trend. Each row corresponds to a trend with a value of *λ* for all the scenarios with different combination of *n*_01_/*n*_0_={0.25,0.5,0.75} and *n*_22_={*n*_02_,*n*_0_,2*n*_0_}. The values with an order of magnitude of -4 are set to zero, as we also obtain such a small number for the unbiased estimators when there is no trend in our simulation, which is due to the Monte Carlo simulation error.

**Table 1:** The maximum (median) absolute bias of the estimated difference in mean responses of the newly added treatment and the control arm when there is a trend. Values with -4 order of magnitude are set to zero

*λ*	*WLS*_*all*_	*WLS*_*s*2_	*M*_*a*1_	*M*_*a*2_	*M*_*b*1_	*M*_*b*2_
Linear trend						
2%	0.007 (0.005)	0 (0)	0.001 (0)	0 (0)	0 (0)	0 (0)
4%	0.015 (0.010)	0 (0)	0.001 (0)	0 (0)	0 (0)	0 (0)
6%	0.022 (0.015)	0 (0)	0.001 (0)	0 (0)	0 (0)	0 (0)
8%	0.030 (0.020)	0 (0)	0.001 (0)	0 (0)	0 (0)	0 (0)
Step trend						
2%	0.015 (0.010)	0 (0)	0.007 (0.004)	0 (0)	0.010 (0.005)	0 (0)
4%	0.030 (0.020)	0 (0)	0.015 (0.008)	0 (0)	0.019 (0.010)	0 (0)
6%	0.045 (0.030)	0 (0)	0.023 (0.012)	0 (0)	0.029 (0.015)	0 (0)
8%	0.060 (0.040)	0 (0)	0.031 (0.016)	0 (0)	0.038 (0.019)	0 (0)

When there is a positive trend in the simulation, we find that the estimates obtained from *WLS*_*s*2_,*M*_*a*2_ and *M*_*b*2_ respectively are unbiased. For those obtained from *WLS*_*all*_, the magnitude of the bias is larger when there is a step trend than when there is a linear trend. This is because the presence of a positive trend inflates the estimate of the overall mean responses of the control treatment while the mean responses of the newly added treatment only have a trend that is similar to the mean responses of the second stage control treatment.

For those obtained from *M*_*a*1_ and *M*_*b*1_, the estimated parameter is almost unbiased when there is a linear trend but is biased when there is a step trend. This shows that adjustment with a linear term may not always yield accurate estimate especially when the structure of a trend is not linear.

### Marginal power in the presence of a trend

The first row of plots in Figs. 2 and 3 show the marginal power of rejecting *H*_02_ when there is no trend; other rows of plots correspond to different magnitudes of *λ* when there is a linear and a step trend respectively. The *x*-axis of the plots indicates the value of *n*_22_ and *n*_01_/*n*_0_. Each column of plots has a different range of *y*-axis due to having different values of *n*_22_ when the new arm is added to the on-going trial. As expected, the marginal power obtained from *WLS*_*s*2_ is similar to that from *M*_*a*2_: the blue line (with ×) superimposes the red line (with *red*△).

We see that the marginal power obtained from *WLS*_*all*_ is the highest and from *M*_*b*2_ is the lowest among all the comparators. However, the marginal power obtained from *WLS*_*all*_ is inflated when there is a positive trend. From our simulation, we find that relative difference in the inflated power to the non-inflated power is larger when *n*_22_=550 compared to when *n*_22_=1100. The reason could be when *n*_22_ is small (relative to the fixed *n*_01_), stage one control responses deviate more on average from the responses in stage two, which leads to a larger bias when using all the control data and hence increases the average number of rejected hypotheses.

On the other hand, the marginal power obtained from *M*_*a*1_ and *M*_*b*1_ respectively when there is a linear trend remains the same as when there is no trend (i.e. compare the black lines and cyan lines respectively across the row of plots in Fig. 2); but it is inflated when there is a step trend. The magnitude of the inflation increases with the values, *λ*, of the step trend (see Fig. 3).

We find that the marginal power obtained from *WLS*_*s*2_,*M*_*a*2_ and *M*_*b*2_ respectively remains unchanged when there is a trend. This is not surprising as the impact of a positive trend in the mean responses of the newly added arm and the second stage control arm are of similar magnitude, which is being removed when the difference in mean responses is considered.

### Type one error rate in the presence of a trend

**Fig. 4:**
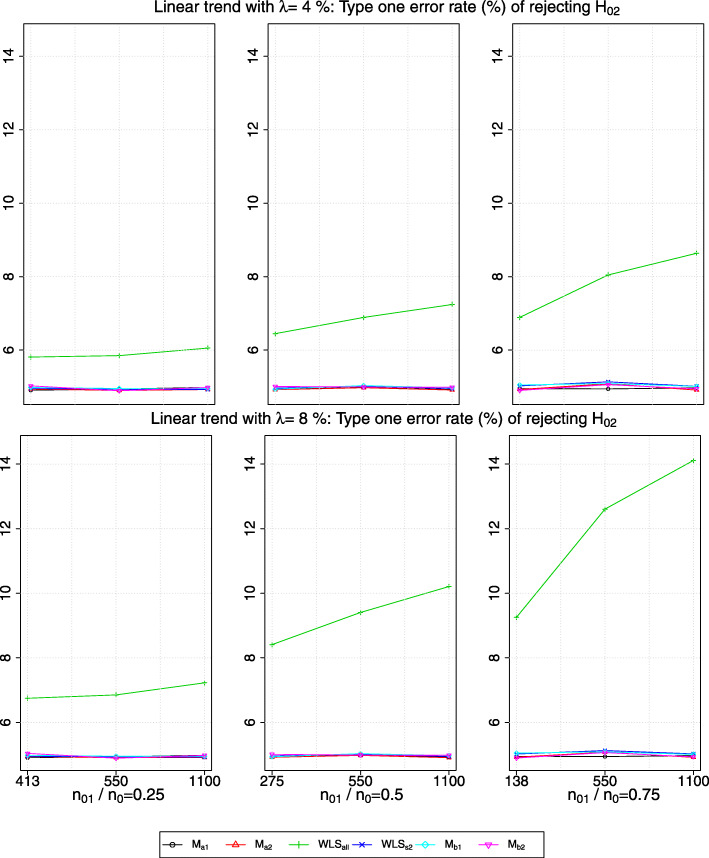
The type one error rate of rejecting *H*_02_ in the presence of a linear trend with a magnitude of *λ*>0 standard deviation (row-wise); *x*-axis indicates the value of *n*_22_ and the timing of adding the new arm

**Fig. 5:**
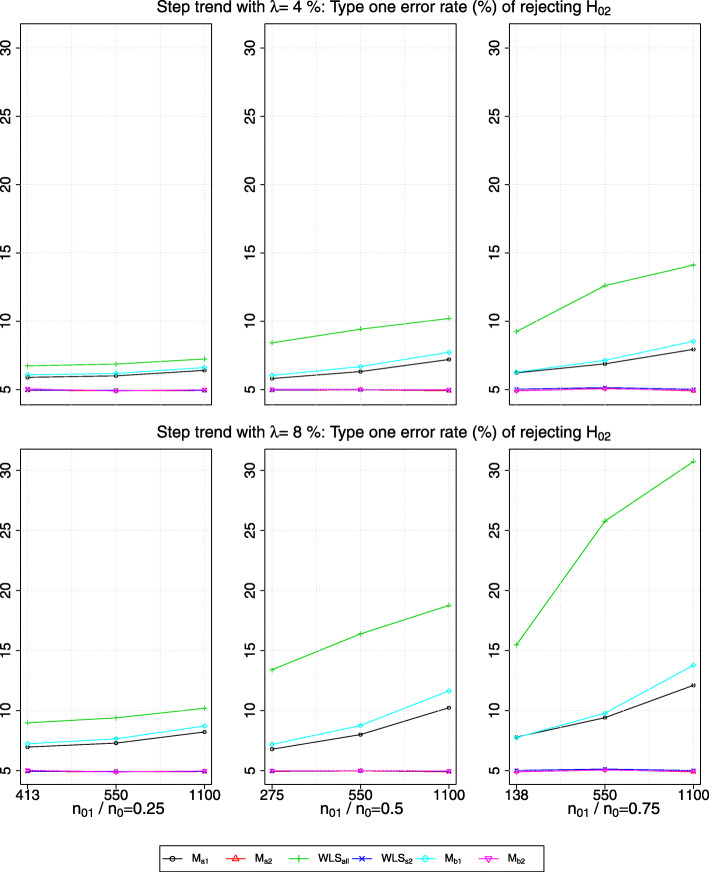
The type one error rate of rejecting *H*_02_ in the presence of a step trend with a magnitude of *λ*>0 standard deviation (row-wise); *x*-axis indicates the value of *n*_22_ and the timing of adding the new arm

Figures 4 and 5 show the type one error rate of rejecting *H*_02_ in the presence of a linear trend and of a step trend respectively, when *δ*_1_=*δ*_2_=0. A subtle difference in the type one error rate obtained from *WLS*_*s*2_ and *M*_*b*2_ respectively can be seen in the plots.

We observe that the presence of a step trend would lead to a larger inflation in the type one error rate obtained from *WLS*_*all*_ than when there is a linear trend with the same *λ*, i.e. from comparing the green line (with *green*+ sign) in Fig. 4 to those in Fig. 5. Besides, the magnitude of inflation is the largest when both the values of *n*_01_/*n*_0_ and *λ* are the largest. Having larger *n*_22_ also causes more inflation in the type one error rate given the same *n*_01_/*n*_0_. This may be because, given the same magnitude of the bias, large *n*_22_ makes the denominator of the test statistics smaller, and hence increases the number of hypothesis rejections.

For the type one error rate obtained from *M*_*a*1_ and *M*_*b*1_, we see a similar finding to that of the marginal power. That is, when there is a linear trend, the type one error rate is maintained within the Monte Carlo simulation error, whereas it is inflated when there is a step trend (see the black line and cyan line respectively in Fig. 5). We find that the type one error rate obtained from *WLS*_*s*2_,*M*_*a*2_ and *M*_*b*2_ respectively is maintained within the Monte Carlo simulation error when there is a trend.

### rMSE in the presence of a trend

The plots of rMSE are presented in Additional file 1: Figures 1 and 2 in the supplement. When there is a positive trend, we find that *M*_*b*2_ has the highest rMSE, which remains the same across all the considered values of *λ*. This is not surprising as the least amount of the data is used. The rMSE values obtained from *WLS*_*s*2_ and *M*_*a*2_ also remain unchanged when there is a trend.

When there is a linear trend, the rMSE obtained from *WLS*_*all*_ increases with *λ* while remaining smaller than that from *M*_*a*1_ and *M*_*b*1_ (which remain unchanged across the linear trends). However, when there is a step trend, the rMSE obtained from these approaches are inflated; the rMSE obtained from *WLS*_*all*_ is higher than that from *M*_*a*1_ when *λ*≥4*%*. Nevertheless, across all the considered scenarios, the rMSE obtained from *M*_*a*1_ is always smaller than the approaches that use less data, i.e. *M*_*a*2_,*M*_*b*1_,*M*_*b*2_, and *WLS*_*s*2_.

These findings highlight that even though the presence of a small trend would lead to a small bias in the estimation, the quality of the estimate computed using all the control data can be better than that computed using only the concurrent control data. This does not hold for larger trends.

### Inference about the initial treatment

For the inference about the initial treatment, we find that the bias, type one error rate and power of rejecting *H*_01_ are maintained at the same levels as those when there is no trend in the simulation. This finding is observed for all the model-based approaches. This may be due to using the restricted randomization procedure within each stage of the trial in the simulation, which ensures that not all *n*_*ks*_ patients are being enrolled to a particular arm *k* during the early or the late phase of the trial. As a result, the average impact of a trend on the stage-wise mean responses of the initial treatment and of the control treatment are similar, which are then being subtracted when the difference in the mean responses is considered. This observation is consistent with the finding of Ryeznik and Sverdlov [17] who compare different randomization procedures in the context of the standard ANOVA *F*-test.

## Discussion

When there is no trend, the advantages of including the non-concurrent control data are greater when the new arm is added at a later time point. Our simulation studies show that given the same magnitude of *λ*>0, the presence of a step trend affects the validity of the inference more than that of a linear trend when all the control data are used in the analysis of the new arm. We also examine that using a linear regression model adjusting for a linear term may lead to spurious findings when there is a non-linear trend, though the rMSE of the corresponding estimate can be lower than other approaches that use only the concurrent control data. This represents a caveat for including non-concurrent control data in the analysis plan as it is impossible to know the structure of a trend at the planning stage of a trial. After data collection, one can only test the presence of a trend using some quality control techniques such as control charts and CUSUM analysis, see for example a review by Noyez [31]. The analogy between clinical trials and industrial processes is becoming clearer especially when the trial has a long enrolment period. [32*–*38]

This work has highlighted the impact of a trend when the variance of the observations is known. We emphasise that not all methods assume known variance. We conjecture that the presented results would hold when an estimated variance is unbiased, i.e. the estimate is robust to the presence of a trend. Otherwise, the over- or under-estimated variance would cause the test statistics to be biased and hence provide a spurious result [28]. We also note that when there is a negative trend, i.e. *λ*<0, the type one error and the marginal power will be deflated, i.e. smaller than the nominal value. This is a direct consequence of a trend that causes responses to be smaller than the true values as the trial progresses.

All things considered, we would recommend that at the design stage of phase III trials, sample size calculations assume only concurrent control information will be used. When the magnitude of a trend is small, we find including the non-concurrent control data in the analysis can improve the efficiency of estimating the parameter of interest. However there could be an inflation (or deflation) in the type one error rate and the marginal power of testing the corresponding hypothesis unless the positive (or negative) trend has a magnitude of less than 0.5% of the standard deviation (based on simulation results not presented here). This finding is similar to the finding of Kopp-Schneider *et al* [39] for the situation where external information is used in the inference of clinical trials: power gain is not possible when requiring a strict type one error rate control. When the recruitment to all arms do not finish simultaneously, the control data can be separated into before and after adding an arm and those after the initial treatment arms finish recruitment. In this case, the presence of a trend across the three stages can be tested before utilising all the control data to increase the precision of the inference.

We acknowledge that an inflated power is not an issue if we are confident that the intervention is effective. However, using the trial result for other purposes may lead to negative consequences since an inflated power could mean that the estimated effect size is likely to be higher than it should. For example, doing a cost-effectiveness analysis using the estimated effect size may over-estimate the value of the intervention due to the presence of a positive trend. Future work could review real trials that add arms and explore the presence of trends and their structure in the data. One can also investigate the potential of using non-concurrent control data for the situations where i) there are more than one treatment arm being added to the on-going trial; ii) there are more than two-stages; and iii) adaptive randomization procedures are considered.

## Conclusion

Platform trials can potentially speed up the drug development processes. The feature of continuous recruitment to the control arm may increase the precision of the inference about the newly added interventions in some situations; or reduce the required sample size for the evaluation of the newly added interventions at the cost of having a less stringent control of the error rates. In light of the presence of a potential trend, it is wise to compare the results of including and excluding non-concurrent control data in the analysis of the newly added interventions.

## Supplementary information

**Additional file 1** Figures 1 and 2 show the rMSE when there is a linear and a step trend respectively in the simulation.

## Data Availability

Not applicable.

## Abbreviations

BoS: borrowing of strength
rMSE: root mean squared error
