# Supplementary material for: Including non-concurrent control patients in the analysis of platform trials: is it worth it?
Source: BMC Med Res Methodol. 2020 Jun 24;20:165. doi: 10.1186/s12874-020-01043-6 (PMC7315495; doi:10.1186/s12874-020-01043-6)
Supplement: Supplementary file 1 — Additional file 1 Figures 1 and 2 show the rMSE when there is a linear and a step trend respectively in the simulation. [file 12874_2020_1043_MOESM1_ESM.pdf]

Additional File 1

Figures 1 and 2 show the rMSE when there is a linear and a step trend respectively in the simulation.

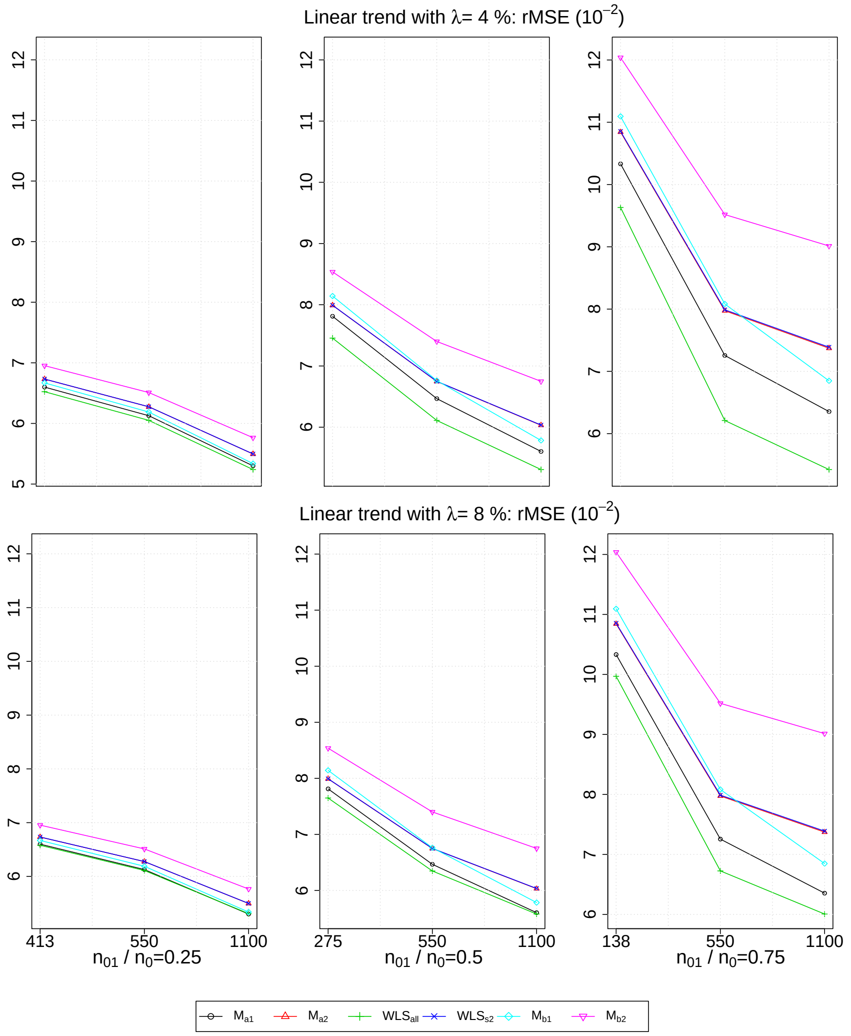

Figure 1 rMSE when there is a linear trend with a magnitude of $\lambda > 0$ standard deviation. The x-axis indicates the value of n_{22} and the timing of adding the new arm.

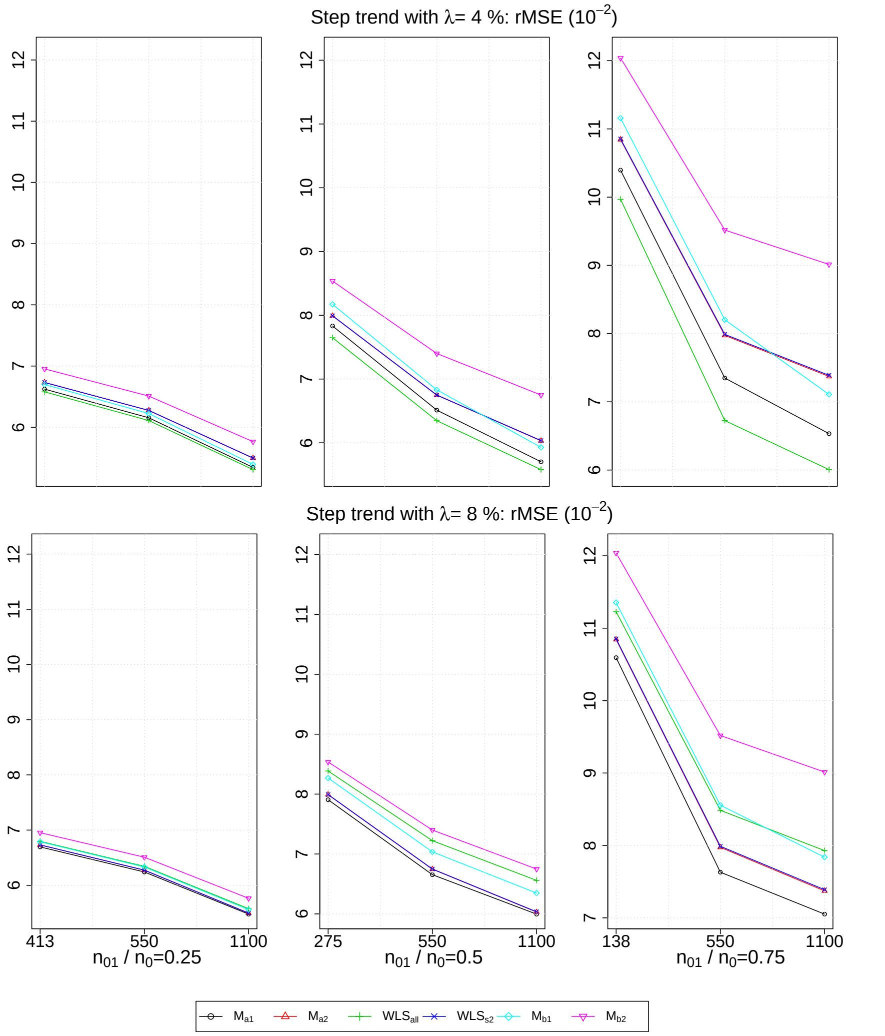

Figure 2 rMSE when there is a step trend with a magnitude of $\lambda > 0$ standard deviation. The x-axis indicates the value of n_{22} and the timing of adding the new arm.
